# Assessing Associations Between COVID-19 Symptomology and Adverse Outcomes After Piloting Crowdsourced Data Collection: Cross-sectional Survey Study

**DOI:** 10.2196/37507

**Published:** 2022-12-06

**Authors:** Natalie Flaks-Manov, Jiawei Bai, Cindy Zhang, Anand Malpani, Stuart C Ray, Casey Overby Taylor

**Affiliations:** 1 Johns Hopkins University School of Medicine Baltimore, MD United States; 2 Johns Hopkins Bloomberg School of Public Health Baltimore, MD United States; 3 Johns Hopkins Whiting School of Engineering Baltimore, MD United States

**Keywords:** COVID-19, coronavirus, symptoms, symptomology, crowdsourcing, adverse outcomes, data quality

## Abstract

**Background:**

Crowdsourcing is a useful way to rapidly collect information on COVID-19 symptoms. However, there are potential biases and data quality issues given the population that chooses to participate in crowdsourcing activities and the common strategies used to screen participants based on their previous experience.

**Objective:**

The study aimed to (1) build a pipeline to enable data quality and population representation checks in a pilot setting prior to deploying a final survey to a crowdsourcing platform, (2) assess COVID-19 symptomology among survey respondents who report a previous positive COVID-19 result, and (3) assess associations of symptomology groups and underlying chronic conditions with adverse outcomes due to COVID-19.

**Methods:**

We developed a web-based survey and hosted it on the Amazon Mechanical Turk (MTurk) crowdsourcing platform. We conducted a pilot study from August 5, 2020, to August 14, 2020, to refine the filtering criteria according to our needs before finalizing the pipeline. The final survey was posted from late August to December 31, 2020. Hierarchical cluster analyses were performed to identify COVID-19 symptomology groups, and logistic regression analyses were performed for hospitalization and mechanical ventilation outcomes. Finally, we performed a validation of study outcomes by comparing our findings to those reported in previous systematic reviews.

**Results:**

The crowdsourcing pipeline facilitated piloting our survey study and revising the filtering criteria to target specific MTurk experience levels and to include a second attention check. We collected data from 1254 COVID-19–positive survey participants and identified the following 6 symptomology groups: abdominal and bladder pain (Group 1); flu-like symptoms (loss of smell/taste/appetite; Group 2); hoarseness and sputum production (Group 3); joint aches and stomach cramps (Group 4); eye or skin dryness and vomiting (Group 5); and no symptoms (Group 6). The risk factors for adverse COVID-19 outcomes differed for different symptomology groups. The only risk factor that remained significant across 4 symptomology groups was influenza vaccine in the previous year (Group 1: odds ratio [OR] 6.22, 95% CI 2.32-17.92; Group 2: OR 2.35, 95% CI 1.74-3.18; Group 3: OR 3.7, 95% CI 1.32-10.98; Group 4: OR 4.44, 95% CI 1.53-14.49). Our findings regarding the symptoms of abdominal pain, cough, fever, fatigue, shortness of breath, and vomiting as risk factors for COVID-19 adverse outcomes were concordant with the findings of other researchers. Some high-risk symptoms found in our study, including bladder pain, dry eyes or skin, and loss of appetite, were reported less frequently by other researchers and were not considered previously in relation to COVID-19 adverse outcomes.

**Conclusions:**

We demonstrated that a crowdsourced approach was effective for collecting data to assess symptomology associated with COVID-19. Such a strategy may facilitate efficient assessments in a dynamic intersection between emerging infectious diseases, and societal and environmental changes.

## Introduction

COVID-19 represents a global public health concern [1-3]. While extensive measures are being implemented to control the outbreak, the high speed of transmission makes collecting data needed to inform clinical management and public health planning a challenge. Efficiently collecting high-quality data to characterize disease severity enables accurate information to be disseminated in a timely manner for such planning.

To understand and predict the adverse health outcomes in patients affected by COVID-19, many scientific efforts studying sociodemographic, clinical, and symptomatic risk factors are underway. Findings from those efforts, however, are not all consistent, with conflicting evidence on the risk factors associated with adverse COVID-19 outcomes [4-6]. Furthermore, infected people have reported a wide range of symptoms, from asymptomatic to severe illness [2-12]. Common symptoms include fever, cough, fatigue, shortness of breath, and loss of the sense of smell or taste, and less frequent symptoms are gastrointestinal and neurological symptoms [4-6,13-16]. Although there has been a concerted effort to describe patients’ symptoms [7,17,18], there is no evidence yet as to whether symptoms differ between people with different characteristics, such as chronic diseases and demographic backgrounds [19,20]. As individual symptoms cannot predict COVID-19 adverse outcomes [21], knowledge of a patient’s profile of symptoms (ie, symptomology) holds promise to improve estimations of the risk of adverse outcomes [22].

A crowdsourcing model is a useful way to rapidly collect information in the context of the COVID-19 pandemic [23,24]. Recent work to classify different types of crowdsourcing used to tackle the COVID-19 crisis [23] found that the most common configuration to deal with information and knowledge management problems was open crowdsourcing (described as a one-to-many configuration with potentially unlimited contributors, and without any form of preselection). Most initiatives falling under this category, however, demonstrated a desire to locate and assemble information. The *COVID Near You* website [25], for example, uses crowdsourced data to visualize maps to identify current and potential pandemic hotspots. An important emphasis for crowdsourced data, however, is to collect high-quality data. Indeed, the risk of bias can be great when building COVID-19 diagnosis and prognosis prediction models trained on small or low-quality data sets. The majority of COVID-19 prediction models to date, for example, show a high risk of bias (n=226, 97%) [26].

To eliminate substandard crowd data submissions, we used a “crowdsourcing via a broker” strategy with broker services that allowed for filtering participants and their responses, and testing data quality before finalizing the crowdsourcing data collection strategy. We chose to use the Amazon Mechanical Turk (MTurk) crowdsourcing platform that provides filtering mechanisms via setting qualifications [27-30]. Through the MTurk platform, entities known as “requesters” can hire independent contractors, known as “workers,” to perform a wide variety of remote jobs, known as “human intelligence tasks” (HITs). A worker’s reputation is indicated by their HIT acceptance rate [30]. The emphasis on obtaining good-quality data through setting qualifications, however, has created some concern about “superworkers.” These are experienced and very active MTurk workers to whom researchers often target survey distribution. This oversampling from experienced workers can lead to an issue of worker nonnaivete as workers are frequently exposed to common methods in research studies. Recent research shows that nonsuperworkers can also produce high-quality data [31], and our strategy thus incorporated a pilot phase with broad inclusion criteria according to experience qualifications. Rather than defaulting to experienced workers, the pilot data collection allowed us to determine what filtering criteria were best suited to our needs.

In this paper, we describe (1) a pipeline to enable data quality and population representation checks in a pilot setting prior to deploying the final survey to MTurk workers, (2) an assessment of COVID-19 symptomology among MTurk worker survey respondents who reported a previous positive COVID-19 result, and (3) an assessment of the associations of symptomology groups and underling chronic conditions with adverse outcomes due to COVID-19.

## Methods

### Study Design and Instrument

This was a cross-sectional study. We developed 2 web-based surveys using Qualtrics. One survey was for individuals (ie, individual survey) who indicated a self-reported positive test for COVID-19, and another survey was for individuals whose relatives (ie, family survey), living in the same house, tested positive for COVID-19. We hosted both surveys on MTurk between August and December 2020. To improve the quality of data collection through MTurk and to make our study sample more representative of the target population, we followed the best practices suggested by Young et al [30].

A few restrictions were implemented to exclude certain survey responses from the final data analysis. First, only those participants who provided an existing COVID-19 test type (nasal/throat/blood/sputum) answer in response to our screening question could continue with the survey. Second, participants could fill the survey only once for themselves (individual survey) and for 1 family member (family survey). Third, a quality control question was included during the questionnaire, which stated, “Do not answer this question (Please click NEXT to go to the next question).” If the question was answered, the survey responses were excluded from the data analyses. Fourth, in the family survey, we asked the participants about their confidence level in their responses regarding their family member. Responses with low reported confidence were excluded from the final analyses.

### Ethical Considerations

This study was judged as imposing only minimal risks on participants and was determined to be exempt research by the Johns Hopkins University (IRB00248053) Institutional Review Board.

### Variables and Definitions

The web-based surveys had 5 blocks as follows (Multimedia Appendix 1 and Multimedia Appendix 2):

1. *Introduction and screening*: Introduction to the aim of the study, including the estimated amount of time needed to complete the survey, the compensation amount, information about the voluntary nature of the survey, and instructions on not filling out the questionnaire more than once.
2. *Symptoms of COVID-19*: We asked participants to select all the symptoms that they experienced following a COVID-19 infection.
3. *Adverse outcomes of COVID-19*: We asked questions about hospitalizations related to COVID-19 and connection to a mechanical ventilator.
4. *Medical history*: We asked questions about background medical conditions, smoking status, and influenza vaccine status in a previous season.
5. *Demographic characteristics*: Participants reported on their age, sex they were assigned at birth, race, ethnicity, last year’s income (monthly and yearly), and the highest level of education.

Survey measures were from the Johns Hopkins University COVID-19 community response survey guidance toolkit that draws from multiple sources [32]. An additional data source we used beyond the toolkit to compile COVID-19 symptoms was Twitter [18,32,33].

### Recruitment

#### Population

The inclusion criteria for this study were individuals living in the United States, adults (aged 18 years or older), and MTurk workers with a self-reported positive COVID-19 result. For the family survey, the participants could complete the survey for 1 family member, even if the family member, who lived in the same household, was below 18 years old. Thus, the target population of this study was COVID-19 patients living in the United States and having sufficient skills to use the MTurk platform. The participants were compensated according to a standard minimum wage and our estimate of completion time (about 5-10 min).

#### Crowdsourcing Pipeline

Before posting the final survey to MTurk, we conducted a pilot from August 5, 2020, to August 14, 2020, to assess the quality of responses among workers with different levels of experience. The pilot analysis stratified the worker sample into the following 3 experience groups: those who previously completed 100-499 HITs, 500-999 HITs, and 1000+ HITs. First, a worker would complete a qualification test asking them to verify that they or a family member tested positive for COVID-19. If qualified, the worker could then start the MTurk HIT that included a link to the 26-question web-based Qualtrics survey. The first part of the survey was a screening question (age ≥18 years) and comprehension check. In response to the comprehension check, if a worker selected an invalid COVID-19 test type (eg, urine test), they could not continue the survey.

For those passing the screening test, responses were labeled as “high quality” according to the following criteria: sufficient time taken (threshold of more than 60 s); matching codes and IDs between Qualtrics and MTurk; each code being associated with only 1 worker; and worker had not taken the survey previously (ie, nonduplicate response). A worker’s response was included in the “high-quality” group if they passed all of these criteria.

Separately, we assessed “nonduplicate responses.” A nonduplicate response indicates that the respondent completed the survey only once. This criterion was considered under the assumption that workers who attempted to complete the survey multiple times to receive more compensation did not read through survey instructions carefully, and thus, they may provide lower quality responses than those who attempted to complete the survey once.

The general characteristics of age, sex, race, education, and income were extracted and compared among experience groups. Chi-square analysis was conducted to evaluate if there was a significant difference between experience groups in the number of high-quality and nonduplicate responses. Findings from this analysis were used to refine our filtering criteria in the final crowdsourcing pipeline.

### Statistical Analysis

#### Outcomes

We assessed the following 2 primary adverse outcomes related to COVID-19: hospital admission due to COVID-19 and use of mechanical ventilation during admission.

#### Statistical Analyses

We used descriptive statistics to characterize the total cohort of participants. Bivariate analyses, using Pearson χ^2^ tests, were performed to assess differences in participant characteristics between those hospitalized and those not hospitalized, and between those who needed mechanical ventilation during admission and those who did not need mechanical ventilation. We then fitted multivariate logistic regression models to identify the association of COVID-19 symptoms with hospitalization and mechanical ventilation due to COVID-19, adjusted for sociodemographic characteristics and comorbid conditions. Thereafter, hierarchical cluster analysis was conducted to search for patterns based on COVID-19 symptoms. The similarity measure was cosine similarity, and the linkage method was *Ward* minimum variance. To describe clusters, we calculated frequencies of the risk factors for each cluster of symptoms. We then developed logistic regression models for hospitalization and mechanical ventilation as outcomes, using symptomology groups as risk factors. Finally, we developed a logistic regression model for each symptomology group to identify the significant risk factors for hospitalization among individuals with different symptomology. All analyses were performed using R version 3.6.2 (R Foundation for Statistical Computing).

### Validation Assessment

To validate our findings, we performed a comparison with existing systematic review or meta-analysis papers that assessed symptoms as risk factors for COVID-19 adverse outcomes. Articles for which the analyses occurred prior to our data collection were selected for comparison.

For each article and this study, individual symptoms were checked for being reported as (1) a significant risk factor for an adverse outcome (“yes”) and (2) a nonsignificant risk factor for an adverse outcome (“no”). We also noted if a symptom was not assessed (“NA”). When synthesizing findings across studies, if we found a statistically significant association between an adverse outcome and a symptom that was not studied by others, we labeled it “New.” If there was agreement between this study and at least one other study in identifying a symptom as a risk factor (significant or nonsignificant), we labeled it “1.” Symptoms we did not assess were labeled “NA.”

## Results

### Pilot Findings

Pilot survey data were collected from 259 respondents who passed both the qualification test and the screening questions, and of these, 147 (56.8%) were considered to have “high quality” responses. For the experience groups 100-499, 500-999, and 1000+ HITs, the proportions of high-quality responses were 58% (48/83), 43% (41/95), and 72% (58/81), respectively (Table 1). There was no significant difference between the experience groups for obtaining high-quality responses (*P*=.14). There was, however, a significant difference between the groups for nonduplicate responses (*P*<.001). Comparisons of demographic characteristics across all experience groups among MTurk workers are shown in Multimedia Appendix 3.

Two modifications were made to our crowdsourcing pipeline following the pilot. First, we included only workers with 500+ prior HITs in our final filtering criteria. Given the differences in nonduplicate responses between groups, we reasoned that for tasks requiring a higher cognitive ability, workers with 500+ HITs may provide more high-quality responses than those with 100-499 HITs. Second, we added an attention check question to the Qualtrics survey (ie, “don’t answer this question”).

**Table 1:** Comparison of the approval rates and percentage of quality responses out of approved responses between the different experience groups.

Variable	Experience group			*P* value
	100-499 HITs^a^ , n/N (%)	500-999 HITs, n/N (%)	1000+ HITs, n/N (%)	
High-quality responses	48/83 (58)	41/95 (43)	58/81 (72)	0.135
High-quality and nonduplicate responses	10/48 (21)	41/41 (100)	49/58 (85)	<0.001

^a^HIT: human intelligence task.

### Survey Responses

**Figure 1:**
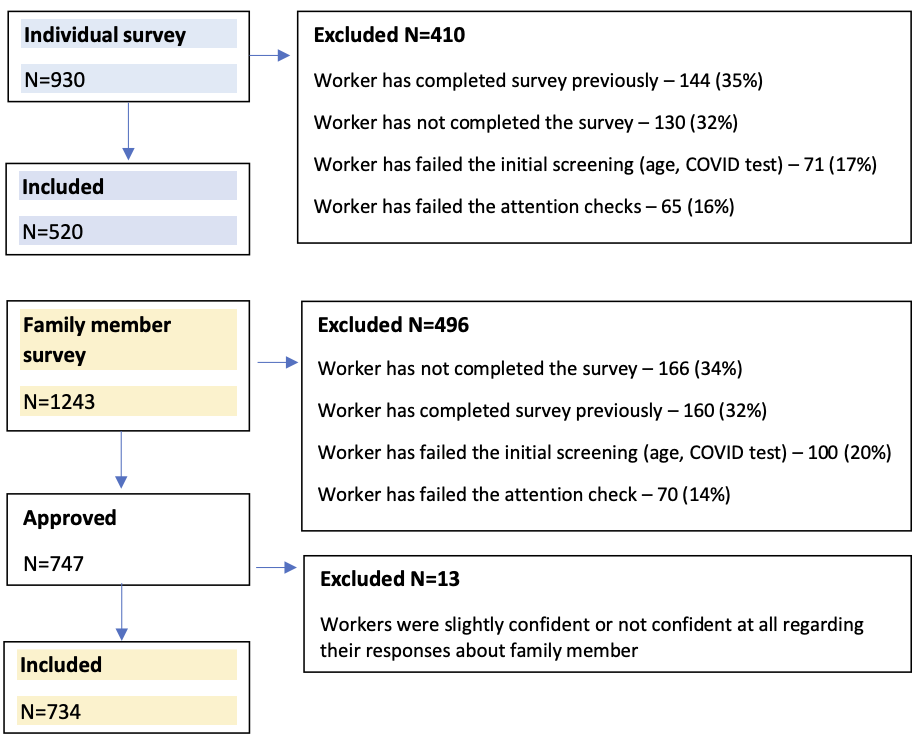
Study Inclusion and Exclusion of Amazon Mechanical Turk Worker Responses.

After implementing our final crowdsourcing pipeline, we collected data from 930 individual surveys and 1243 family surveys; however, data from 410 individual surveys and 496 family surveys were excluded (late August to December 31, 2020). The reasons for exclusion were completion of the survey previously, noncompletion of the survey, initial screening failure for age or comprehension check, and attention check failure (Figure 1). Thus, we finally collected data from 1267 eligible COVID-19–positive participants, and of these, 520 were from individual surveys and 747 were from family surveys. Thirteen participants were further excluded as they were either only slightly confident (n=12) or not confident at all (n=1) regarding their responses in the family survey. Thus, data from 1254 surveys were analyzed. The average time required to complete the general survey was 5.5 minutes.

Regarding family survey respondents, 68.3% (501/734) provided answers about a first-degree family member, 25.7% (189/734) provided answers about a second-degree family member, and only 6.0% (44/734) provided answers about a third-degree relative. There were no statistically significant differences in characteristics or outcomes between the individual respondents and the persons the respondents completed the family survey for, except for age (Multimedia Appendix 4). Therefore, the analysis presented here combined data from both surveys.

### Demographic Characteristics

Over 90% (1159/1254, 92.4%) of the participants were up to 65 years old, and only 1.2% (15/1254) were less than 18 years old. Moreover, 52.0% (652/1254) were male, 81.2% (1018/1254) were white, 79.5% (997/1254) were not Hispanic or Latino, 68.4% (858/1254) had a bachelor’s degree or any postgraduate degree, 14.4% (180/1254) had yearly income of US $75,000 or more, 39.6% (496/1254) were smokers, and 46.8% (587/1254) had an influenza vaccine in the last season (Multimedia Appendix 5). Eight responders mentioned “passed away” in the family survey. As reflected in our sample, the MTurk worker population tended to be younger than the overall US population, with household incomes below that of the average US population [27].

### Findings From Assessing Individual Symptoms Associated With Adverse COVID-19 Outcomes

#### Hospitalization

Overall, 47.6% (597/1254) of participants were hospitalized due to COVID-19. Bivariate analysis showed statistically significant differences between hospitalized and nonhospitalized COVID-19 participants for most demographic factors, except gender (Multimedia Appendix 5). Chronic conditions, including depression, hypertension, asthma, alcohol disorder, anemia, weight loss, ulcer, lung/respiratory disease, bladder problems, bowel disease, and angina, were associated with more COVID-19 hospitalizations (Multimedia Appendix 6). COVID-19 symptoms associated with higher risk for hospitalization were cough with sputum, sneezing, abdominal pain, vomiting, confusion, bladder pain, dry eyes, dry skin, skin rash, and seizure (Multimedia Appendix 7).

From the logistic regression analysis of the total study population (Multimedia Appendix 8), we found statistically significant associations between the following participant characteristics and COVID-19 hospitalization compared with baseline: being in any age group over 24 years; having a bachelor’s degree (odds ratio [OR] 2.57, 95% CI 1.43-4.66); smoking every day, smoking some days, or past smoking with quitting less than a year ago (OR 2.06, 95% CI 1.18-3.61; OR 3.41, 95% CI 2.2-5.33; and OR 3.39, 95% CI 1.88-6.24, respectively); and influenza vaccine in the last season (OR 3.09, 95% CI 2.18-4.41). Chronic conditions associated with higher risk for hospitalization were depression (OR 1.77, 95% CI 1.18-2.67), asthma (OR 3.83, 95% CI 2.22-6.78), diabetes (OR 2.66, 95% CI 1.46-4.96), and bladder problems (OR 5.51, 95% CI 1.28-27.13). COVID-19 symptoms associated with higher risk for hospitalization were abdominal pain (OR 2.02, 95% CI 1.15-3.59), bladder pain (OR 3.20, 95% CI 1.25-9.3), cough with sputum (OR 2.60, 95% CI 1.77-3.86), fever with a temperature over 100.4°F (OR 1.50, 95% CI 1.04-2.17), and shortness of breath (OR 2.75, 95% CI 1.8-4.23).

#### Mechanical Ventilation

Overall, 66.8% (399/597) of hospitalized participants were connected to a mechanical ventilator (31.8% of all participants). There were 11 hospitalized participants from the family survey whose mechanical ventilation use was unknown to the survey respondents, and these participants were not included in the subsequent mechanical ventilation analysis. Smoking every day (OR 3.51, 95% CI 1.45-9.1), influenza vaccine in the last season (OR 3.65, 95% CI 2.29-5.89), loss of appetite (OR 2.07, 95% CI 1.09-4.02), tiredness and fatigue (OR 2.36, 95% CI 1.04-5.44), and vomiting (OR 2.68, 95% CI 1.3-5.71) were significantly associated with higher risk for mechanical ventilation (Multimedia Appendix 8).

### Findings From Assessing COVID-19 Symptomology

**Figure 2:**
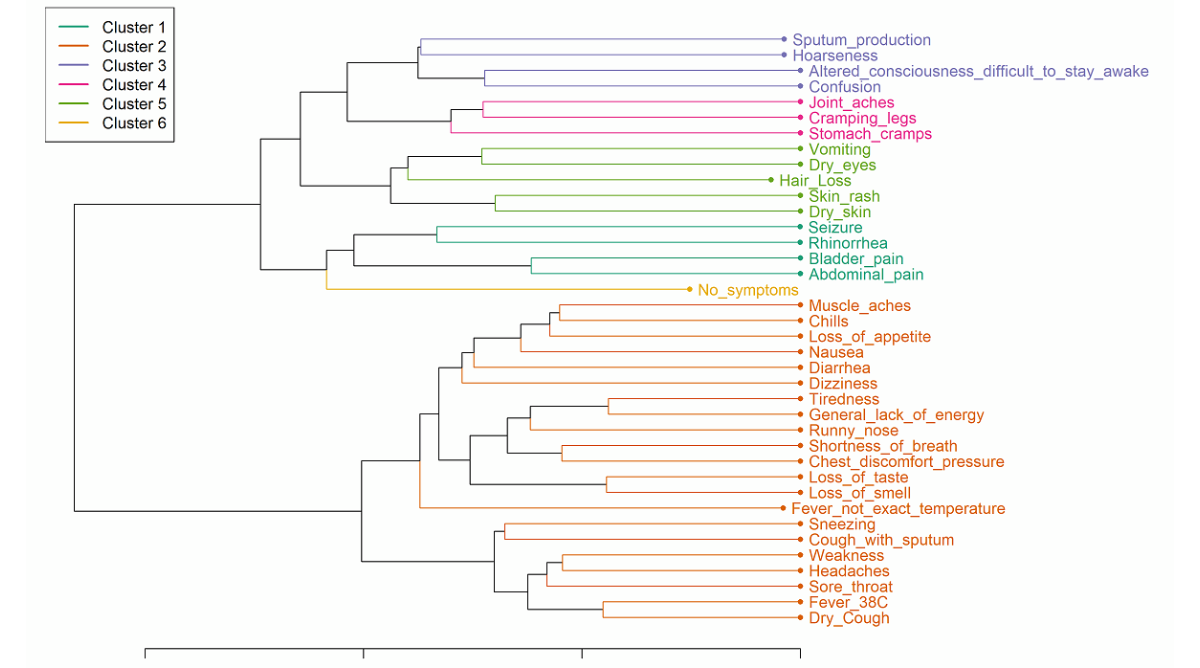
Dendrogram for COVID-19 symptom clusters.

We identified the following 6 symptomology groups using hierarchical cluster analysis (Figure 2): Group 1, abdominal and bladder pain; Group 2, flu-like symptoms (loss of smell/taste/appetite); Group 3, hoarseness and sputum production; Group 4, joint aches and stomach cramps; Group 5, skin or eye dryness and vomiting; and Group 6, no symptoms. We found sociodemographic and clinical differences between the symptomology groups (Table 2).

The flu-like symptoms group (Group 2) mostly represented the general study population. The abdominal and bladder pain group (Group 1) and the skin or eye dryness group (Group 5) had the highest hospitalization frequencies (153/227, 67.4% and 134/196, 68.4%, respectively). Both groups were characterized by a lower chance of high income (19/227, 8.4% and 21/196, 10.7%, respectively), more smoking (121/227, 53.3% and 102/196, 52.0%, respectively), and more influenza vaccinations (144/227, 63.4% and 102/196, 52.0%, respectively). The group with abdominal and bladder pain symptoms (Group 1) had higher proportions of Hispanic participants (82/227, 36.1%), asthma patients (64/227, 28.2%), alcohol disorder patients (64/227, 28.2%), and anemia patients (54/227, 23.8%). The group with skin or eye dryness (Group 5) had higher proportions of patients with depression (64/196, 32.7%), diabetes (28/196, 14.3%), weight loss (27/196, 13.8%), and ulcers (25/196, 12.8%). The group with joint aches and stomach cramps (Group 4) had lower proportions of hospitalization (65/158, 41.1%) and mechanical ventilation (31/158, 47.7%). Compared with the general study population, the asymptomatic group (Group 6) was younger (age 18-44 years; 70/85, 82.4%), had more males (54/85, 63.5%), had less white participants (60/85, 70.6%), had less Hispanic participants (7/85, 8.2%), had more participants with a high income (18/85, 21.2%), had less smokers (26/85, 30.6%), had less influenza vaccinations reported (30/85, 35.3%), had a higher proportion of participants with no chronic conditions (45/85, 52.9%), and had a very low risk for hospitalization (12/85, 14.1%).

**Table 2:** Descriptive characteristics of COVID-19 symptomology groups.

Characteristic		Group 1 (abdominal and bladder pain) (N=227), n (%)	Group 2 (flu-like symptoms) (N=1139), n (%)	Group 3 (hoarseness, sputum production) (N=144), n (%)	Group 4 (joint aches, stomach cramps) (N=158), n (%)	Group 5 (skin or eye dryness) (N=196), n (%)	Group 6 (no symptoms) (N=85), n (%)
**Hospitalization**		153 (67.4)	621 (54.5)	79 (54.9)	65 (41.1)	134 (68.4)	12 (14.1)
	Mechanical ventilation (among hospitalized patients)	112 (73.2)	366 (58.9)	46 (58.2)	31 (47.7)	86 (64.2)	10 (83.3)
**Demographic characteristics**							
	Male gender	107 (47.1)	588 (51.6)	64 (44.4)	87 (55.1)	91 (46.4)	54 (63.5)
	Age 18-44 years	148 (65.2)	727 (63.8)	82 (56.9)	94 (59.5)	133 (67.9)	70 (82.4)
	Age ≥45 years	76 (33.5)	379 (33.3)	54 (37.5)	58 (36.7)	57 (29.1)	15 (17.6)
	White race	186 (81.9)	929 (81.6)	118 (81.9)	134 (84.8)	168 (85.7)	60 (70.6)
	Hispanic or Latino ethnicity	82 (36.1)	230 (20.2)	23 (16.0)	27 (17.1)	43 (21.9)	7 (8.2)
	US $75,000 or more yearly income	19 (8.4)	162 (14.2)	20 (13.9)	28 (17.7)	21 (10.7)	18 (21.2)
	Smoking	121 (53.3)	450 (39.5)	53 (36.8)	45 (28.5)	102 (52.0)	26 (30.6)
	Flu vaccination	144 (63.4)	533 (46.8)	62 (43.1)	69 (43.7)	102 (52.0)	30 (35.3)
**Chronic conditions**							
	Depression	38 (16.7)	285 (25.0)	35 (24.3)	37 (23.4)	64 (32.7)	12 (14.1)
	Obesity	32 (14.1)	165 (14.5)	35 (24.3)	37 (23.4)	29 (14.8)	6 (7.1)
	Asthma	64 (28.2)	156 (13.7)	29 (20.1)	19 (12.0)	29 (14.8)	6 (7.1)
	Alcohol or substance use disorder	64 (28.2)	128 (11.2)	19 (13.2)	17 (10.8)	25 (12.8)	7 (8.2)
	Diabetes, uncomplicated	12 (5.3)	116 (10.2)	15 (10.4)	19 (12.0)	28 (14.3)	1 (1.2)
	Mental illness	36 (15.9)	98 (8.6)	23 (16.0)	22 (13.9)	17 (8.7)	4 (4.7)
	Migraines	23 (10.1)	81 (7.1)	15 (10.4)	23 (14.6)	15 (7.7)	4 (4.7)
	Weight loss	20 (8.8)	76 (6.7)	14 (9.7)	15 (9.5)	27 (13.8)	3 (3.5)
	Anemia	54 (23.8)	70 (6.1)	12 (8.3)	13 (8.2)	22 (11.2)	3 (3.5)
	High cholesterol	7 (3.1)	69 (6.1)	14 (9.7)	20 (12.7)	9 (4.6)	3 (3.5)
	Ulcer	10 (4.4)	68 (6.0)	5 (3.5)	11 (7.0)	25 (12.8)	3 (3.5)
	No chronic condition	34 (15.0)	264 (23.2)	23 (16.0)	31 (19.6)	29 (14.8)	45 (52.9)

#### Symptomology Groups Associated With Adverse COVID-19 Outcomes

Our findings from the logistic regression models, using symptomology groups as risk factors for adverse COVID-19 outcomes and adjusted for all sociodemographic characteristics and comorbid conditions, showed the following 3 groups associated with hospitalization: abdominal and bladder pain group (Group 1; OR 1.5, 95% CI 1.01-2.34); flu-like symptoms group (Group 2; OR 3.33, 95% CI 1.97-5.79); and skin or eye dryness group (Group 5; OR 1.63, 95% CI 1.07-2.52). No symptomology group was associated with a high risk for mechanical ventilation (Table 3).

**Table 3:** Associations between COVID-19 symptomology groups and adverse COVID-19 outcomes.

Symptomology group^a^	Hospitalization^b^				Mechanical ventilation^b^			
	OR^c^	95% CI	*P* value	OR		95% CI	*P* value	
Abdominal and bladder pain group (Group 1)	1.54	1.01-2.34	.04	1.12		0.66-1.92	.68	
Flu-like symptoms group (Group 2)	3.33	1.97-5.79	<.001	0.17		0.04-0.54	.01	
Hoarseness and sputum production group (Group 3)	1.51	0.92-2.48	.10	1.09		0.57-2.11	.80	
Joint aches and stomach cramps group (Group 4)	0.56	0.35-0.88	.01	0.54		0.27-1.07	.08	
Skin or eye dryness group (Group 5)	1.63	1.07-2.52	.02	1.25		0.76-2.05	.39	

^a^Group 6 (no symptoms) is excluded.

^b^Multivariate logistic models adjusted for sociodemographic characteristics and comorbid conditions.

^c^OR: odds ratio.

#### Risk Factors for COVID-19 Hospitalization Among Symptomology Groups

**Figure 3:**
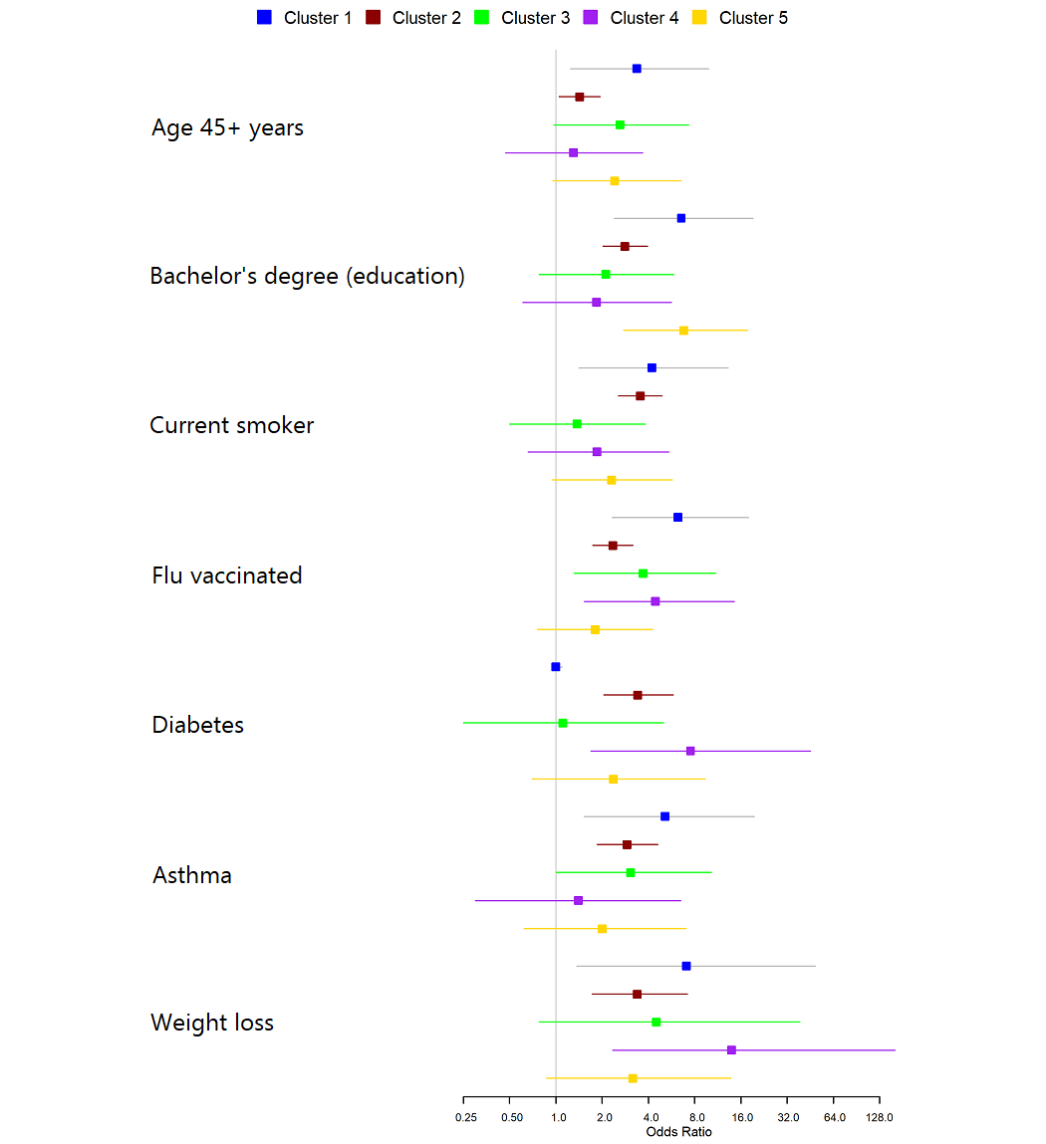
Risk factors for hospitalization among individuals in different symptomology groups.

Finally, we developed 5 logistic regression models for symptomology groups to compare the risk factors for COVID-19 hospitalization among those groups (asymptomatic participants were excluded from this analysis). The results of those models are presented as a forest plot of significant variables in at least one symptomology group (Figure 3). The risk factors differed between participants from different symptomology groups. The only risk factor that was significant for 4 out of 5 groups was influenza vaccine in the last season (Group 1: OR 6.22, 95% CI 2.32-17.92; Group 2: OR 2.35, 95% CI 1.74-3.18; Group 3: OR 3.7, 95% CI 1.32-10.98; Group 4: OR 4.44, 95% CI 1.53-14.49). Smoking (OR 4.22, 95% CI 1.42-13.26) and asthma (OR 5.14, 95% CI 1.53-19.56) were significant risk factors for hospitalization in the abdominal and bladder pain group (Group 1). Weight loss was a risk factor in the joint aches and stomach cramps group (Group 4; OR 13.9, 95% CI 2.34-161.64) and in the abdominal and bladder pain group (Group 1; OR 7.05, 95% CI 1.37-49.01). Diabetes was a risk factor in the joint aches and stomach cramps group (Group 4; OR 7.5, 95% CI 1.69-45.28).

### Findings From the Validation Assessment

A comparison of our findings with those of other studies can be found in Multimedia Appendix 9. At the time of our analysis, we found 3 systematic review or meta-analysis studies mapping the association of symptoms with the risk of adverse outcomes of COVID-19 [19-21].

We found agreement between this study and previous studies for 18 symptoms, 6 of which were associated with adverse outcomes (abdominal pain, cough, dyspnea/shortness of breath, fever, fatigue, and vomiting). In addition, we assessed 14 symptoms that were not previously studied by others, 6 of which were associated with adverse outcomes (bladder pain, dry eyes, dry skin, loss of appetite, seizure, and skin rash).

## Discussion

### Principal Findings

Our results identified individual symptoms and behaviors associated with COVID-19 adverse outcomes. Among these, some were well-known and some were new. We also identified 6 symptomology groups, with 3 groups showing statistically significant associations with COVID-19 outcomes. Furthermore, the findings of this work increase our understanding of the MTurk population and show that with precautionary measures, high-quality data can be obtained.

Well-known single COVID-19 symptoms identified (ie, abdominal pain, cough, fever, and shortness of breath) were associated with hospitalization [5,6]. Less common symptoms identified, such as bladder pain, eye dryness, and skin dryness were also associated with adverse COVID-19 outcomes. We provided additional validation of our findings by comparing the results with the findings of systematic review and meta-analysis studies. The individual symptoms we identified as being associated with adverse COVID-19 outcomes were consistent with the symptoms in those studies.

Our analysis of chronic conditions and associations with COVID-19 adverse outcomes showed that patients with preexisting asthma, diabetes, depression, and bladder problems were at high risk for hospitalization, similar to the findings in previous studies. Although previous studies have shown an increased risk of severe COVID-19 among people with obesity [34], our study did not find a significant increase in the risk of hospitalization among obese people. This result may be due to the participants in our sample being younger than those in other studies, resulting in a weaker link between obesity and chronic diseases that are the actual drivers of COVID-19 severity.

When studying behaviors influencing adverse COVID-19 outcomes, like previous studies, we found that smoking increased the risk of severe COVID‐19 outcomes [35-37]. Current smokers and past smokers who quit less than a year ago had a higher risk of hospitalization, and every day smokers also had a higher risk for mechanical ventilation. Our finding showing an effect of influenza vaccination on adverse outcomes contradicts the findings in some other studies. For example, it has been previously reported that influenza vaccination could be considered a protective factor again severe cases of COVID-19 infection [38,39]. Our data, however, suggested that COVID-19–positive respondents who were vaccinated against influenza in Autumn 2019 had higher odds of hospitalization and mechanical ventilation after adjusting for demographic factors, chronic conditions, and COVID-19 symptoms, as the influenza vaccination status might be associated with preexisting comorbidities and a person’s demographics. This is not an isolated finding as others have reported that there is a positive association between influenza vaccination rates and COVID-19 death rates [40], that influenza vaccination coverage in a country is a risk factor associated with higher infection rates of COVID-19 [41], and that there is a need to investigate the potential impact of influenza vaccination on COVID-19 risk and severity [42].

In addition to studying individual symptoms and behaviors, this study identified 6 COVID-19 symptomology groups by cluster analysis and assessed their associations with adverse outcomes of the disease. Three symptomology groups (flu-like symptoms, abdominal and bladder pain symptoms, and eye and skin dryness symptoms) were highly associated with a high risk for hospitalization. While the characteristics of respondents in the flu-like symptoms group were similar to the characteristics of the general population, the abdominal and bladder pain group included survey respondents who had lower income, and were more likely to have smoked and to be influenza vaccinated. They also tended to have chronic conditions, such as asthma and anemia, and alcohol disorder. The survey respondents in the eye and skin dryness group were generally older and had a greater possibility of being white. They were also more likely to have smoked and to be influenza vaccinated. This group also had a very high percentage of survey respondents with depression, diabetes, and ulcers.

Characterizing patients according to clusters using artificial intelligence devices and machine learning is a pioneering method in a variety of infectious and noninfectious diseases. The use of scientific methods to identify clusters of patients with similar characteristics and specific disease risks might improve awareness of heterogeneity in symptomology, and may enable targeted interventions to reduce disease severity. Other studies of COVID-19 disease trajectories have been able to identify vulnerable population clusters that could benefit from specific health resources, and have provided insights for public health targets for managing the pandemic [43,44]. One previous study identified 3 symptomatic groups and 1 asymptomatic group among COVID-19 patients [43]. However, that study did not analyze the associations between the symptomology groups and COVID-19 outcomes. Our analysis of 6 symptomatic groups found that the risk factors for COVID-19 adverse outcomes differed between participants from the different symptomology groups. For the asymptomatic COVID-19 group, other studies have shown that asymptomatic carriers account for 15% to 60% of the infected population and play a key role in disease transmission [45]. Adding to our understanding of asymptomatic carriers, our findings indicated that the asymptomatic symptomology group had a low percentage of hospitalization; a high percentage of young non-Hispanic men with high income; and a low percentage of people with chronic conditions, smoking, and influenza vaccination. These characteristics add to those described in a review study of asymptomatic COVID-19 carriers’ characteristics that found young age alone to be a significant factor for having no symptoms [46-48]. Another study of Mexican outpatients found a lower frequency of smokers and influenza vaccination among asymptomatic responders [43].

The percentage of those connected to a mechanical ventilator among hospitalized patients may seem high in our study (61.8%); however, the management of patients hospitalized with COVID-19 has changed considerably over the course of the pandemic. More than half of the study population had been hospitalized, and two-thirds of them were on ventilators. Since the survey was conducted in the first months of the COVID-19 pandemic, many people who got sick with COVID-19 were hospitalized and then connected to a mechanical ventilator. Over time, fewer people with COVID-19 were hospitalized, and among those who were hospitalized, only patients with more severe disease were put on ventilators. Other studies have also shown a high percentage (68%) of ventilator use among hospitalized COVID patients [49].

This work also showed that with precautionary measures to ensure high-quality data collection, a crowdsourcing model can be used to collect data to characterize symptomology for COVID-19 diagnosis and prognosis. There are many studies assessing health data on MTurk as a source of high-quality and rapidly collected data, and it has demonstrated good reliability [30,50,51]. However, to improve data quality on MTurk, there are recommendations to include workers with an “approval rate” above 95% and keep the “number of HITs approved” to at least 100 [30,31,51]. Prior studies have not investigated data quality from workers by comparing survey responses of 3 experience levels (according to the number of HITs approved) in a pilot study. By launching a pilot study, we found no difference in the approval rate of workers from different experience groups; thus, all could provide adequate data to satisfy the basic approval criterion. For specific tasks requiring higher cognitive ability, however, workers with more experience may provide higher quality data. In our case, we found that those with 500+ HITs submitted fewer duplicate responses than those with 100-499 HITs. While this may exacerbate the superworker issue, the tradeoff of quality data for the use of more experienced workers may be necessary depending on the task. To provide additional validation of our findings, we compared the findings of individual symptoms associated with COVID-19 to the findings of other researchers and identified many concordant findings.

### Limitations

A major limitation of this study was the self-reported data, which can be less reliable than physiological assessments. Our crowdsourced approach, however, allowed for reaching many participants, which helped mitigate the noise, and the fast data collection process was helpful during this pandemic. In addition, during this pandemic, many risk factors of COVID-19 were discovered through social media and other self-reported surveys [52-56]. To use those data sources, crowdsourced practices are emerging in research fields such as *infodemiology* (defined as collecting and analyzing data in real time through an electronic medium with the aim to inform public health decision makers) [57-59]. Another growing field is *digital epidemiology*, in which researchers are using internet data for epidemiological purposes [60-62]. The techniques of capturing relevant real-world data are promising but need to be further developed to meet the possible public health challenges in the future. Second, some of our findings warrant further validation. The risk factors first reported in our study, such as bladder pain symptoms and eye or skin dryness symptoms, need to be more extensively studied so that they can be used in clinical assessments. Furthermore, the influence of influenza vaccination on COVID-19 adverse outcomes should be further investigated as it appears now that humans will have to co-exist with both diseases for a long time even after this pandemic.

### Conclusions

Our work demonstrated that a crowdsourced approach was effective for collecting data to assess the symptomology associated with COVID-19. Conducting a pilot study to assess data quality and population representation facilitated refining the filtering criteria for our final data collection strategy. We validated our approach by comparing the findings from assessing individual symptoms associated with COVID-19 to those identified by others and found highly concordant results. In our assessment of symptomology groups, we discovered that the bladder pain and skin or eye dryness groups had a high risk of COVID-19 hospitalization. Given these findings, we believe that a crowdsourcing strategy, such as the one proposed here, should be considered by others for quick and cost-effective assessments in a rapidly changing spectrum of infectious diseases, and societal and environmental factors.

## Appendix

Multimedia Appendix 1 Individual survey of COVID-19 symptoms.

Multimedia Appendix 2 Family survey of COVID-19 symptoms.

Multimedia Appendix 3 Comparison of demographic characteristics across all experience groups among approved Amazon Mechanical Turk workers.

Multimedia Appendix 4 Descriptive characteristics of participants in the individual and family surveys.

Multimedia Appendix 5 Demographic characteristics of the study participants.

Multimedia Appendix 6 Chronic condition characteristics of the study participants.

Multimedia Appendix 7 COVID-19 symptom characteristics of the study participants.

Multimedia Appendix 8 Associations between symptoms and adverse COVID-19 outcomes adjusted for sociodemographic factors and chronic conditions (multivariate logistic regression).

Multimedia Appendix 9 Comparison of our findings with those of systematic review and meta-analysis studies regarding the association between COVID-19 symptoms and adverse outcomes.

## Abbreviations

HIT: human intelligence task
MTurk: Amazon Mechanical Turk
OR: odds ratio
