# Supplementary material for: Assessing Associations Between COVID-19 Symptomology and Adverse Outcomes After Piloting Crowdsourced Data Collection: Cross-sectional Survey Study
Source: JMIR Form Res. 2022 Dec 6;6(12):e37507. doi: 10.2196/37507 (PMC9746676; doi:10.2196/37507)
Supplement: Multimedia Appendix 1 [file formative_v6i12e37507_app1.docx]

**Multimedia Appendix 1.** Individual survey of COVID-19 symptoms.

1. What type of COVID-19 test did you take? **(Select all that apply)**

- Nasopharyngeal swab (Nasal)
- Oropharyngeal swab (Throat)
- Blood test
- Saliva test (sputum)
- Urine test
- I did not get tested
- Other ________________________________________________

2. Are you 18 years or older?

- Yes
- No

3. Please select the symptoms you experienced following a Covid-19 infection **(Select all that apply)**^1^

*NOTE:* ***No symptoms****should be selected if none were present*

- Abdominal pain
- Bladder pain
- Chest discomfort, tightness or pressure
- Chills
- Confusion
- Cough with sputum
- Cramping legs
- Diarrhea
- Dizziness
- Dry Cough
- Dry skin
- Dry eyes
- Fever > 100.4 F or > 38 C
- Fever but do not know exact temperature (no thermometer)
- General lack of energy or malaise
- Headaches
- Hair Loss
- Hoarseness
- Joint aches
- Loss of ability to smell
- Loss of ability to taste
- Loss of appetite
- Muscle aches
- Nausea
- Rhinorrhea
- Runny or stuffy nose
- Seizure
- Shortness of breath
- Skin rash
- Sneezing
- Sore throat
- Sputum production
- Stomach cramps
- Tiredness or Fatigue
- Vomiting
- Weakness
- Altered consciousness or feeling like it was difficult to stay awake
- Other symptoms ________________________________________________
- **No Symptoms**

4. Of all the symptoms you reported, **which one symptom was the most bothersome** (i.e., severe) to you?^1^

- Abdominal pain
- Bladder pain
- Chest discomfort, tightness or pressure
- Chills
- Confusion
- Cough with sputum
- Cramping legs
- Diarrhea
- Dizziness
- Dry Cough
- Dry skin
- Dry eyes
- Fever > 100.4 F or > 38 C
- Fever but do not know exact temperature (no thermometer)
- General lack of energy or malaise
- Headaches
- Hair Loss
- Hoarseness
- Joint aches
- Loss of ability to smell
- Loss of ability to taste
- Loss of appetite
- Muscle aches
- Nausea
- Rhinorrhea
- Runny or stuffy nose
- Seizure
- Shortness of breath
- Skin rash
- Sneezing
- Sore throat
- Sputum production
- Stomach cramps
- Tiredness or Fatigue
- Vomiting
- Weakness
- Altered consciousness or feeling like it was difficult to stay awake
- Other symptom_______________________________________________

5. How bothersome or distressful was that symptom?^1^

- Not at all
- A little bit
- Somewhat
- Quite a bit
- Very much

6. Have you been hospitalized for COVID-19 or because you had difficulty breathing or a respiratory infection?^1^

- Yes
- No

7. How many days have you been hospitalized?

- 1-2 days
- 3-4 days
- 5-6 days
- 7-10 days
- 11-15 days
- more than 15 days

8. Have you been connected to a ventilator machine due to respiratory failure?

- Yes
- No

9. What is your status now?^1^

- You recovered and are symptom free
- You are feeling better but not completely recovered
- You are not feeling better

10. Do you smoke tobacco products (e.g., cigarettes, cigars, pipes)?

- Yes, every day
- Yes, some days
- Past smoker, I quit less than a year ago
- Past smoker, I quit more than a year ago
- Never smoked on permanent basis

11. Have you had a flu vaccine this season?

- Yes
- No

12. Have you had a tuberculosis vaccine within the past 10 years?

- Yes
- No
- Do not remember

13. Do not answer this question (Please click “NEXT” to go to the next question)

- Yes
- Maybe
- No

14. Have you EVER been told by a doctor or other health professional that you have any of the following medical conditions?^2^(**Select all that apply)**
*NOTE:* ***None*** *should be selected if none of the listed conditions were ever present*

- Alcohol or substance use disorder
- Anemia
- Asthma
- Autoimmune problems
- Bladder Problems
- Bleeding Disease (Coagulopathy)
- Bowel Disease
- Cancer
- Cardiac arrhythmia
- Chronic kidney disease
- Chronic obstructive pulmonary disease (COPD)
- Congestive heart failure
- Depression
- Diabetes, uncomplicated
- Diabetes, complicated
- Drug abuse
- Heart Pain/Angina
- Hepatitis B virus (HBV)
- Hepatitis C virus (HCV)
- High Blood Pressure
- High Cholesterol
- HIV
- Hypertension
- Lung/Respiratory Disease
- Mental Illness
- Migraines
- Obesity
- Osteoporosis
- Psychoses
- Paralysis
- Other neurological disorders
- Reflux/GERD
- Renal failure
- Seizures/Convulsions
- Severe Allergy
- Stroke/CVA of the Brain
- Thyroid Problems
- Tuberculosis (TB)
- Ulcer
- Valvular heart disease
- Weight loss
- Other mental health condition___________________________
- Other chronic condition_________________________________
- None

[The following question will only show when certain conditions are selected]

What type of cancer do you have? (**Select all that apply)**

- Blood (Leukemia)
- Breast
- Colon
- Lung
- Lymphoma
- Pancreas
- Prostate
- Skin (Melanoma)
- Other________________________________________________

Please specify the cancer status:

- Ongoing
- Past
- Recent Remission

Please specify the status of Lung/Respiratory Disease

- Past
- Current

Please specify the status of Hepatitis C virus (HCV)

- Past
- Current

Please specify the status of Asthma

- Past
- Current

Please specify the status of Cardiac arrhythmia

- Past
- Current

Please specify the status of Heart Pain/Angina

- Past
- Current

[End of question 14]

15. Are you currently taking any medications for any of **these conditions**?^2^

- Yes
- No

16. Are you currently taking any medications for **any other** health or medical conditions?^2^

- Yes
- No

17. Please list which medications you are taking regularly

________________________________________________________________

________________________________________________________________

________________________________________________________________

18. Have you made arrangements to get your medication refill/s?^2^

- No
- You have been able to arrange for some medication refills but not all
- You are waiting to hear from your physician on how to refill medications
- Yes, home delivery
- Yes, you will be picking up from the pharmacy
- Yes, someone will be picking up your medications for you

19. Since the COVID-19 pandemic (March 1, 2020), have you needed to postpone any medical procedures?^2^

- Yes
- No

20. In the past month, have you missed any scheduled appointments with any health care provider?^2^

- Yes
- No
- Don't know
- Refused to answer

21. In the past month, have you missed taking any medications?^2^

- Yes
- No
- Don't know
- Refused to answer

22. What sex were you assigned at birth, on your original birth certificate?^3^

- Male
- Female

23. What is your age?

________________________________________________________________

24. What is your race?^3^ (**Select all that apply**)

- White
- Black/African American
- Asian American
- Native American/American Indian or Alaska Native
- Native Hawaiian or other Pacific Islander
- Other

25. What is your ethnicity?^3^

- Hispanic or Latino
- Not Hispanic or Latino

26. What was your income last year (in 2019) from all sources before taxes?^3^
 This includes all income from both formal and informal employment.
 Answers show both monthly and yearly incomes. (Choose one)

- Monthly income: $0 to $833; Yearly income: $0 to $9,999
- Monthly income: $834 to $1,250; Yearly income: $10,000 to $14,499
- Monthly income: $1,251 to $2,082; Yearly income: $15,000 to $24,999
- Monthly income: $2,083 to $2,916; Yearly income: $25,000 to $34,999
- Monthly income: $2,917 to $4,167; Yearly income: $35,000 to $49,999
- Monthly income: $4,168 to $6,249; Yearly income: $50,000 to $74,999
- Monthly income: $6,250 or more; Yearly income: $75,000 or more
- Don't know
- Refuse to Answer

27. What is the highest level of education you completed?^3^

- Never attended school
- Grades 1 through 8
- Grades 9 through 11 / Some high school
- Grade 12/Completed high school or GED
- Some college, Associates Degree, or Technical Degree
- Bachelor's Degree
- Any post graduate studies
- Don't Know
- Refuse to Answer

Citations:

1. COVID-19 COMMUNITY RESPONSE SURVEY GUIDANCE - Modul 4: COVID-19 Symptoms and Testing Experience
2. COVID-19 COMMUNITY RESPONSE SURVEY GUIDANCE - Modul 5: Comorbidities and Care Engagement
3. COVID-19 COMMUNITY RESPONSE SURVEY GUIDANCE - Modul1: Demographics

https://www.phenxtoolkit.org/toolkit_content/PDF/JHU_C4WARD.pdf
