# Supplementary material for: Assessing Associations Between COVID-19 Symptomology and Adverse Outcomes After Piloting Crowdsourced Data Collection: Cross-sectional Survey Study
Source: JMIR Form Res. 2022 Dec 6;6(12):e37507. doi: 10.2196/37507 (PMC9746676; doi:10.2196/37507)
Supplement: Multimedia Appendix 2 [file formative_v6i12e37507_app2.docx]

**Multimedia Appendix 2.** Family survey of COVID-19 symptoms.

1. What type of COVID-19 test did your family member take? **(Select all that apply)**

- Nasopharyngeal swab (Nasal)
- Oropharyngeal swab (Throat)
- Blood test
- Saliva test (sputum)
- Urine test
- Did not get tested
- Other _______________________________________________

2. Are you 18 years or older?

- Yes
- No

3. What is your relationship to the family member?^3^

- First-degree relative (e.g., parent, spouse, sibling, children)
- Second-degree relative (e.g., grandparent, grandchild, aunts, uncles, nephews, nieces)
- Third degree relative (e.g., great-grandparent, great grandchildren, great uncles/aunts)
- Other relative/Unknown relationship

4. What sex was your family member assigned at birth, on the original birth certificate?^3^

- Male
- Female

5. What is your family member's age?^3^

- 18 - 24
- 25 - 34
- 35 - 44
- 45 - 54
- 55 - 64
- 65 - 74
- 75 - 84
- 85 or older
- less than 18

6. What is **your family member's** race?^3^ (Select all that apply)

- White
- Black/African American
- Asian American
- Native American/American Indian or Alaska Native
- Native Hawaiian or another Pacific Islander
- Other

7. What is your family member's ethnicity?^3^

- Hispanic or Latino
- Not Hispanic or Latino

8. What was **your family member's**income last year (in 2019) from all sources before taxes?^3^
 This includes all income from both formal and informal employment.
 Answers show both monthly and yearly incomes. (Choose one)

- Monthly income: $0 to $833; Yearly income: $0 to $9,999
- Monthly income: $834 to $1,250; Yearly income: $10,000 to $14,499
- Monthly income: $1,251 to $2,082; Yearly income: $15,000 to $24,999
- Monthly income: $2,083 to $2,916; Yearly income: $25,000 to $34,999
- Monthly income: $2,917 to $4,167; Yearly income: $35,000 to $49,999
- Monthly income: $4,168 to $6,249; Yearly income: $50,000 to $74,999
- Monthly income: $6,250 or more; Yearly income: $75,000 or more
- Don't know
- Refuse to Answer

9. What is the highest level of education **your family member** completed?^3^

- Never attended school
- Grades 1 through 8
- Grades 9 through 11 / Some high school
- Grade 12/Completed high school or GED
- Some college, Associates Degree, or Technical Degree
- Bachelor's Degree
- Any post graduate studies
- Don't Know
- Refuse to Answer

10. Please select the symptoms your family member experienced following a Covid-19 infection^1^ **(Select all that apply)**

*NOTE:* ***No symptoms****should be selected if none were present*

- Abdominal pain
- Bladder pain
- Chest discomfort, tightness or pressure
- Chills
- Confusion
- Cough with sputum
- Cramping legs
- Diarrhea
- Dizziness
- Dry Cough
- Dry skin
- Dry eyes
- Fever > 100.4 F or > 38 C
- Fever but do not know exact temperature (no thermometer)
- General lack of energy or malaise
- Headaches
- Hair Loss
- Hoarseness
- Joint aches
- Loss of ability to smell
- Loss of ability to taste
- Loss of appetite
- Muscle aches
- Nausea
- Rhinorrhea
- Runny or stuffy nose
- Seizure
- Shortness of breath
- Skin rash
- Sneezing
- Sore throat
- Sputum production
- Stomach cramps
- Tiredness or Fatigue
- Vomiting
- Weakness
- Altered consciousness or feeling like it was difficult to stay awake
- Other symptoms________________________________________________
- **No Symptoms**

11. Of all the symptoms you reported, **which one symptom was the most bothersome** (i.e., severe) to your family member?^1^

- Abdominal pain
- Bladder pain
- Chest discomfort, tightness or pressure
- Chills
- Confusion
- Cough with sputum
- Cramping legs
- Diarrhea
- Dizziness
- Dry Cough
- Dry skin
- Dry eyes
- Fever > 100.4 F or > 38 C
- Fever but do not know exact temperature (no thermometer)
- General lack of energy or malaise
- Headaches
- Hair Loss
- Hoarseness
- Joint aches
- Loss of ability to smell
- Loss of ability to taste
- Loss of appetite
- Muscle aches
- Myalgia or Fatigue
- Nausea
- Rhinorrhea
- Runny or stuffy nose
- Seizure
- Shortness of breath
- Skin rash
- Sneezing
- Sore throat
- Sputum production
- Stomach cramps
- Tiredness or Fatigue
- Vomiting
- Weakness
- Altered consciousness or feeling like it was difficult to stay awake
- Other symptom ______________________________________________

12. How bothersome or distressful was that symptom for your family member?^1^

- Not at all
- A little bit
- Somewhat
- Quite a bit
- Very much

13. Has your family member been hospitalized for COVID-19 or because he/she had difficulty breathing or a respiratory infection?^1^

- Yes
- No

14. How many days has he/she been hospitalized?

- 1-2 days
- 3-4 days
- 5-6 days
- 7-10 days
- 11-15 days
- more than 15 days
- Do not remember

15. Has he/she been connected to a ventilator machine due to respiratory failure?

- Yes
- No
- Do not remember

16. What is the status of your family member now?^1^

- Recovered and is symptoms free
- He/She is feeling better but not completely recovered
- He/She is not feeling better
- Other __________________

17. Does your family member smoke tobacco products (e.g., cigarettes, cigars, pipes)?

- Yes, every day
- Yes, some days
- Past smoker, quit less than a year ago
- Past smoker, quit more than a year ago
- Never smoked on permanent basis
- Do not remember/Unsure

18. Has your family member had a flu vaccine this season?

- Yes
- No
- Do not remember/Unsure

19. Has your family member had tuberculosis vaccine within the past 10 years?

- Yes
- No
- Do not remember/Unsure

20. Do not answer this question (Please click “NEXT” to go to the next question)

- Yes
- Maybe
- No

21. Please indicate if YOUR FAMILY MEMBER has a history of the following medical conditions^2^ (**Select all that apply)**
*NOTE:* ***None*** *should be selected if none of the listed conditions were ever present*

- Alcohol or substance use disorder
- Anemia
- Asthma
- Autoimmune problems
- Bladder Problems
- Bleeding Disease (Coagulopathy)
- Bowel Disease
- Cancer
- Cardiac arrhythmia
- Chronic kidney disease
- Chronic obstructive pulmonary disease (COPD)
- Congestive heart failure
- Depression
- Diabetes, uncomplicated
- Diabetes, complicated
- Drug abuse
- Heart Pain/Angina
- Hepatitis B virus (HBV)
- Hepatitis C virus (HCV)
- High Blood Pressure
- High Cholesterol
- HIV
- Hypertension
- Lung/Respiratory Disease
- Mental Illness
- Migraines
- Obesity
- Osteoporosis
- Psychoses
- Paralysis
- Other neurological disorders
- Reflux/GERD
- Renal failure
- Seizures/Convulsions
- Severe Allergy
- Stroke/CVA of the Brain
- Thyroid Problems
- Tuberculosis (TB)
- Ulcer
- Valvular heart disease
- Weight loss
- Other mental health condition _______________________________
- Other chronic condition _____________________________________
- None

[The following question will only show when certain conditions are selected]

What type of cancer does your family member have? (**Select all that apply)**

- Blood (Leukemia)
- Breast
- Colon
- Lung
- Lymphoma
- Pancreas
- Prostate
- Skin (Melanoma)
- Other_______________________________________

Please specify the cancer status?

- Ongoing
- Past
- Recent Remission

Please specify the status of Lung/Respiratory Disease

- Past
- Current

Please specify the status of Hepatitis C virus (HCV)

- Past
- Current

Please specify the status of Asthma

- Past
- Current

Please specify the status of Cardiac arrhythmia

- Past
- Current

Please specify the status of Heart Pain/Angina

- Past
- Current

[End of question 21]

22. Is your family member taking any medications for any of these conditions?^2^

- Yes
- No
- Do not know

23. Has your family member made arrangements to get the medication refill/s?^2^

- No
- He/She has been able to arrange for some medication refills but not all
- He/She is waiting to hear from the physician on how to refill medications
- Yes, home delivery
- Yes, he/she will be picking up from the pharmacy
- Yes, someone will be picking up the medications for your family member
- I don’t know

24. Since the COVID-19 pandemic (March 1, 2020), has your family member needed to postpone any medical procedures?^2^

- Yes
- No
- Do not know

25. In the past month, has your family member missed any scheduled appointments with any health care provider?^2^

- Yes
- No
- Don't know
- Refused to answer

26. In the past month, has your family member missed taking any medications?^2^

- Yes
- No
- Don't know
- Refused to answer

27. How confident are you that your responses to this survey are correct?

- Completely confident
- Fairly confident
- Somewhat confident
- Slightly confident
- Not confident at all

Citations:

1. COVID-19 COMMUNITY RESPONSE SURVEY GUIDANCE - Modul 4: COVID-19 Symptoms and Testing Experience
2. COVID-19 COMMUNITY RESPONSE SURVEY GUIDANCE - Modul 5: Comorbidities and Care Engagement
3. COVID-19 COMMUNITY RESPONSE SURVEY GUIDANCE - Modul1: Demographics

https://www.phenxtoolkit.org/toolkit_content/PDF/JHU_C4WARD.pdf
