# Supplementary material for: Assessing Associations Between COVID-19 Symptomology and Adverse Outcomes After Piloting Crowdsourced Data Collection: Cross-sectional Survey Study
Source: JMIR Form Res. 2022 Dec 6;6(12):e37507. doi: 10.2196/37507 (PMC9746676; doi:10.2196/37507)
Supplement: Multimedia Appendix 3 [file formative_v6i12e37507_app3.docx]

**Multimedia Appendix 3.** Comparison of demographic characteristics across all experience groups among approved Amazon Mechanical Turk workers.

| **Number of HITs** | **100-499 HITs** | | **500-999 HITs** | | **1000+ HITs** | | **P-Value** |
| --- | --- | --- | --- | --- | --- | --- | --- |
|  | **n** | **%** | **n** | **%** | **n** | **%** |  |
| **Total** | 47 |  | 41 |  | 58 |  |  |
| **Age (Avg)** | 44 |  | 42 |  | 43 |  | 0.985 |
| **Sex** |  |  |  |  |  |  |  |
| Female | 27 | 57% | 13 | 32% | 27 | 47% | 0.017 |
| Male | 20 | 43% | 28 | 68% | 31 | 53% |  |
| **Race*** |  |  |  |  |  |  |  |
| White | 45 | 96% | 33 | 80% | 44 | 76% | 0.004 |
| Black/African American | 1 | 2% | 1 | 2% | 10 | 17% |  |
| Asian American | 0 | 0% | 3 | 7% | 2 | 3% |  |
| Others | 1 | 2% | 4 | 10% | 2 | 3% |  |
| **Ethnicity**** |  |  |  |  |  |  |  |
| Not Hispanic or Latino | 42 | 89% | 25 | 61% | 40 | 69% | 0.013 |
| Hispanic or Latino | 5 | 11% | 16 | 39% | 18 | 31% |  |
| **Education** |  |  |  |  |  |  |  |
| None or Some College | 8 | 17% | 10 | 24% | 19 | 33% | 0.024 |
| Bachelor's Degree | 23 | 49% | 19 | 46% | 31 | 53% |  |
| Any post graduate studies | 16 | 34% | 12 | 29% | 8 | 14% |  |
| **Yearly income** |  |  |  |  |  |  |  |
| $0 to $34,999 | 16 | 34% | 15 | 37% | 22 | 38% | 0.184 |
| $35,000 or more | 31 | 66% | 24 | 59% | 34 | 59% |  |

* Chi square analysis conducted on groups white and non-white since individual Black/African American, Asian, and Others group sizes are too small

** Removed 100-499 group from chi-square analysis due to small group size
