# Supplementary material for: Assessing Associations Between COVID-19 Symptomology and Adverse Outcomes After Piloting Crowdsourced Data Collection: Cross-sectional Survey Study
Source: JMIR Form Res. 2022 Dec 6;6(12):e37507. doi: 10.2196/37507 (PMC9746676; doi:10.2196/37507)
Supplement: Multimedia Appendix 4 [file formative_v6i12e37507_app4.docx]

**Multimedia Appendix 4.** Descriptive characteristics of participants in the individual and family surveys.

| **Demographic characteristics** | **Individual survey** | | **Family survey** | | **Combined survey** | |
| --- | --- | --- | --- | --- | --- | --- |
|  | **N** | **%** | **N** | **%** | **N** | **%** |
|  | **520** |  | **734** |  | **1254** |  |
| **Sex** |  |  |  |  |  |  |
| Male | 263 | 50.6% | 389 | 53.0% | 652 | 52.0% |
| Female | 257 | 49.4% | 345 | 47.0% | 602 | 48.0% |
| **Age** |  |  |  |  |  |  |
| less then 18 | 0 | 0.0% | 15 | 2.0% | 15 | 1.2% |
| 18-24 | 44 | 8.5% | 56 | 7.6% | 100 | 8.0% |
| 25-34 | 238 | 45.8% | 200 | 27.2% | 438 | 34.9% |
| 35-44 | 133 | 25.6% | 151 | 20.6% | 284 | 22.6% |
| 45-54 | 76 | 14.6% | 131 | 17.8% | 207 | 16.5% |
| 55-64 | 22 | 4.2% | 108 | 14.7% | 130 | 10.4% |
| 65-74 | 7 | 1.3% | 55 | 7.5% | 62 | 4.9% |
| 75-84 | 0 | 0.0% | 14 | 1.9% | 14 | 1.1% |
| 85+ | 0 | 0.0% | 4 | 0.5% | 4 | 0.3% |
| **Race** |  |  |  |  |  |  |
| White | 423 | 81.3% | 595 | 81.1% | 1018 | 81.2% |
| Black/African American | 55 | 10.6% | 67 | 9.1% | 122 | 9.7% |
| Asian American | 21 | 4.0% | 28 | 3.8% | 49 | 3.9% |
| Native American/American Indian or Alaska Native | 5 | 1.0% | 14 | 1.9% | 19 | 1.5% |
| Multiracial/Other | 16 | 3.1% | 30 | 4.1% | 46 | 3.7% |
| **Ethnicity** |  |  |  |  |  |  |
| Not Hispanic or Latino | 414 | 79.6% | 583 | 79.4% | 997 | 79.5% |
| Hispanic or Latino | 106 | 20.4% | 151 | 20.6% | 257 | 20.5% |
| **Yearly income** |  |  |  |  |  |  |
| $0 to $9,999 | 28 | 5.4% | 58 | 7.9% | 86 | 6.9% |
| $10,000 to $14,499 | 22 | 4.2% | 60 | 8.2% | 82 | 6.5% |
| $15,000 to $24,999 | 71 | 13.7% | 70 | 9.5% | 141 | 11.2% |
| $25,000 to $34,999 | 73 | 14.0% | 113 | 15.4% | 186 | 14.8% |
| $35,000 to $49,999 | 114 | 21.9% | 145 | 19.8% | 259 | 20.7% |
| $50,000 to $74,999 | 130 | 25.0% | 149 | 20.3% | 279 | 22.2% |
| $75,000 or more | 79 | 15.2% | 101 | 13.8% | 180 | 14.4% |
| Don't know/Refuse to answer | 3 | 0.6% | 38 | 5.2% | 41 | 3.3% |
| **Education** |  |  |  |  |  |  |
| Grade 12/Completed high school or GED | 23 | 4.4% | 94 | 12.8% | 117 | 9.3% |
| Some college, Associates Degree, or Technical Degree | 72 | 13.8% | 159 | 21.7% | 231 | 18.4% |
| Bachelor's Degree | 319 | 61.3% | 341 | 46.5% | 660 | 52.6% |
| Any post graduate studies | 102 | 19.6% | 96 | 13.1% | 198 | 15.8% |
| Not Completed high school or GED/Don't Know | 4 | 0.8% | 44 | 6.0% | 48 | 3.8% |
| **Smoking status** |  |  |  |  |  |  |
| Never smoked on permanent basis | 186 | 35.8% | 284 | 38.7% | 470 | 37.5% |
| Past smoker, quit less than a year ago | 58 | 11.2% | 56 | 7.6% | 114 | 9.1% |
| Past smoker, quit more than a year ago | 49 | 9.4% | 95 | 12.9% | 144 | 11.5% |
| Yes, some days | 140 | 26.9% | 188 | 25.6% | 328 | 26.2% |
| Yes, every day | 87 | 16.7% | 81 | 11.0% | 168 | 13.4% |
| Don't remember/Unsure | 0 | 0.0% | 30 | 4.1% | 30 | 2.4% |
| **Flu vaccine last year** |  |  |  |  |  |  |
| No | 252 | 48.5% | 328 | 44.7% | 580 | 46.3% |
| Yes | 268 | 51.5% | 319 | 43.5% | 587 | 46.8% |
| Do not remember/Unsure | 0 | 0.0% | 87 | 11.9% | 87 | 6.9% |
| **Hospitalized** |  |  |  |  |  |  |
| Yes | 274 | 52.7% | 383 | 52.2% | 657 | 52.4% |
| No | 246 | 47.3% | 351 | 47.8% | 597 | 47.6% |
| **Mechanical Ventilation** |  |  |  |  |  |  |
| Yes | 180 | 65.7% | 219 | 57.2% | 399 | 60.7% |
| No | 94 | 34.3% | 153 | 39.9% | 247 | 37.6% |
| Do not remember/Unsure | 0 | 0.0% | 11 | 2.9% | 11 | 1.7% |
| **Covid-19 Symptoms** |  |  |  |  |  |  |
| Dry Cough | 220 | 42.3% | 309 | 42.1% | 529 | 42.2% |
| Fever > 100.4 F or > 38 C | 178 | 34.2% | 285 | 38.8% | 463 | 36.9% |
| Loss of taste | 163 | 31.3% | 286 | 39.0% | 449 | 35.8% |
| Sore throat | 146 | 28.1% | 231 | 31.5% | 377 | 30.1% |
| Headaches | 148 | 28.5% | 223 | 30.4% | 371 | 29.6% |
| Muscle aches | 125 | 24.0% | 229 | 31.2% | 354 | 28.2% |
| Cough with sputum | 131 | 25.2% | 204 | 27.8% | 335 | 26.7% |
| Shortness of breath | 117 | 22.5% | 213 | 29.0% | 330 | 26.3% |
| Weakness | 104 | 20.0% | 208 | 28.3% | 312 | 24.9% |
| Runny nose | 105 | 20.2% | 193 | 26.3% | 298 | 23.8% |
| Loss of appetite | 96 | 18.5% | 179 | 24.4% | 275 | 21.9% |
| Sneezing | 116 | 22.3% | 158 | 21.5% | 274 | 21.9% |
| Chills | 92 | 17.7% | 171 | 23.3% | 263 | 21.0% |
| Chest discomfort pressure | 91 | 17.5% | 169 | 23.0% | 260 | 20.7% |
| Nausea | 101 | 19.4% | 159 | 21.7% | 260 | 20.7% |
| Tiredness | 81 | 15.6% | 178 | 24.3% | 259 | 20.7% |
| Loss of smell | 69 | 13.3% | 144 | 19.6% | 213 | 17.0% |
| General lack of energy | 55 | 10.6% | 122 | 16.6% | 177 | 14.1% |
| Abdominal pain | 66 | 12.7% | 105 | 14.3% | 171 | 13.6% |
| Fever not exact temperature | 40 | 7.7% | 79 | 10.8% | 119 | 9.5% |
| Joint aches | 42 | 8.1% | 76 | 10.4% | 118 | 9.4% |
| Vomiting | 44 | 8.5% | 70 | 9.5% | 114 | 9.1% |
| Diarrhea | 43 | 8.3% | 67 | 9.1% | 110 | 8.8% |
| Dizziness | 39 | 7.5% | 71 | 9.7% | 110 | 8.8% |
| Confusion | 34 | 6.5% | 54 | 7.4% | 88 | 7.0% |
| Bladder pain | 39 | 7.5% | 45 | 6.1% | 84 | 6.7% |
| Dry eyes | 33 | 6.3% | 25 | 3.4% | 58 | 4.6% |
| Dry skin | 25 | 4.8% | 21 | 2.9% | 46 | 3.7% |
| Stomach cramps | 20 | 3.8% | 26 | 3.5% | 46 | 3.7% |
| Hoarseness | 11 | 2.1% | 26 | 3.5% | 37 | 3.0% |
| Cramping legs | 15 | 2.9% | 14 | 1.9% | 29 | 2.3% |
| Skin rash | 12 | 2.3% | 17 | 2.3% | 29 | 2.3% |
| Sputum production | 11 | 2.1% | 14 | 1.9% | 25 | 2.0% |
| Altered consciousness difficult to stay awake | 7 | 1.3% | 18 | 2.5% | 25 | 2.0% |
| Rhinorrhea | 9 | 1.7% | 11 | 1.5% | 20 | 1.6% |
| Hair Loss | 10 | 1.9% | 6 | 0.8% | 16 | 1.3% |
| Seizure | 6 | 1.2% | 10 | 1.4% | 16 | 1.3% |
| Other symptoms | 3 | 0.6% | 6 | 0.8% | 9 | 0.7% |
| No symptoms | 48 | 9.2% | 37 | 5.0% | 85 | 6.8% |
