# Supplementary material for: Assessing Associations Between COVID-19 Symptomology and Adverse Outcomes After Piloting Crowdsourced Data Collection: Cross-sectional Survey Study
Source: JMIR Form Res. 2022 Dec 6;6(12):e37507. doi: 10.2196/37507 (PMC9746676; doi:10.2196/37507)
Supplement: Multimedia Appendix 5 [file formative_v6i12e37507_app5.docx]

**Multimedia Appendix 5.** Demographic characteristics of the study participants.

|  |  |  | **Hospitalization** | | | **Mechanical Ventilation** | | |
| --- | --- | --- | --- | --- | --- | --- | --- | --- |
|  |  |  |  | | |  | | |
| **Characteristics** | | **Total** | **No** | **Yes** | **P Value** | **No** | **Yes** | **P Value** |
|  |  | **1,254** | **597 (47.6%)** | **657 (52.4%)** |  | **247 (38.2%)** | **399 (61.8%)** |  |
| **Gender** | Female | 602 (48.0%) | 287 (48.1%) | 315 (47.9%) |  | 118 (47.8%) | 193 (48.4%) |  |
|  | Male | 652 (52.0%) | 310 (51.9%) | 342 (52.1%) | 1.000 | 129 (52.2%) | 206 (51.6%) | 0.947 |
| **Age groups** | 18 - 24 | 100 (8.0%) | 80 (13.4%) | 20 (3.0%) |  | 11 (4.5%) | 9 (2.3%) |  |
|  | 25 - 34 | 438 (34.9%) | 202 (33.8%) | 236 (35.9%) | <0.001 | 99 (40.1%) | 135 (33.8%) | 0.007 |
|  | 35 - 44 | 284 (22.6%) | 129 (21.6%) | 155 (23.6%) |  | 52 (21.1%) | 101 (25.3%) |  |
|  | 45 - 54 | 207 (16.5%) | 81 (13.6%) | 126 (19.2%) |  | 34 (13.8%) | 92 (23.1%) |  |
|  | 55 - 64 | 130 (10.4%) | 58 (9.7%) | 72 (11.0%) |  | 28 (11.3%) | 40 (10.0%) |  |
|  | 65 - 74 | 62 (4.9%) | 28 (4.7%) | 34 (5.2%) |  | 14 (5.7%) | 18 (4.5%) |  |
|  | 75 or older | 18 (1.4%) | 5 (0.8%) | 13 (2.0%) |  | 8 (3.2%) | 4 (1.0%) |  |
|  | less then 18 | 15 (1.2%) | 14 (2.3%) | 1 (0.2%) |  | 1 (0.4%) | 0 (0.0%) |  |
| **Race** | White | 1,018 (81.2%) | 449 (75.2%) | 569 (86.6%) |  | 217 (87.9%) | 345 (86.5%) |  |
|  | Black/African American | 122 (9.7%) | 70 (11.7%) | 52 (7.9%) | <0.001 | 21 (8.5%) | 29 (7.3%) | 0.537 |
|  | Asian American | 49 (3.9%) | 38 (6.4%) | 11 (1.7%) |  | 3 (1.2%) | 7 (1.8%) |  |
|  | Native American/American Indian or Alaska Native | 19 (1.5%) | 10 (1.7%) | 9 (1.4%) |  | 3 (1.2%) | 6 (1.5%) |  |
|  | Multiracial/Other | 46 (3.7%) | 30 (5.0%) | 16 (2.4%) |  | 3 (1.2%) | 12 (3.0%) |  |
| **Ethnicity** | Not Hispanic or Latino | 997 (79.5%) | 506 (84.8%) | 491 (74.7%) |  | 212 (85.8%) | 269 (67.4%) |  |
|  | Hispanic or Latino | 257 (20.5%) | 91 (15.2%) | 166 (25.3%) | <0.001 | 35 (14.2%) | 130 (32.6%) | <0.001 |
| **Yearly income** | $0 to $14,499 | 168 (13.4%) | 85 (14.2%) | 83 (12.6%) |  | 32 (13.0%) | 51 (12.8%) |  |
|  | $15,000 to $34,999 | 327 (26.1%) | 143 (24.0%) | 184 (28.0%) | 0.040 | 61 (24.7%) | 119 (29.8%) | 0.232 |
|  | $35,000 to $49,999 | 259 (20.7%) | 124 (20.8%) | 135 (20.5%) |  | 58 (23.5%) | 75 (18.8%) |  |
|  | $50,000 to $74,999 | 279 (22.2%) | 129 (21.6%) | 150 (22.8%) |  | 46 (18.6%) | 102 (25.6%) |  |
|  | $75,000 or more | 180 (14.4%) | 87 (14.6%) | 93 (14.2%) |  | 43 (17.4%) | 49 (12.3%) |  |
|  | Refuse to answer | 41 (3.3%) | 29 (4.9%) | 12 (1.8%) |  | 7 (2.8%) | 3 (0.8%) |  |
| **Education** | Grade 12/Completed high school or GED | 117 (9.3%) | 75 (12.6%) | 42 (6.4%) |  | 24 (9.7%) | 16 (4.0%) |  |
|  | Some college, Associates Degree, or Technical Degree | 231 (18.4%) | 161 (27.0%) | 70 (10.7%) | <0.001 | 30 (12.1%) | 35 (8.8%) | 0.010 |
|  | Bachelor's Degree | 660 (52.6%) | 252 (42.2%) | 408 (62.1%) |  | 139 (56.3%) | 266 (66.7%) |  |
|  | Any post graduate studies | 198 (15.8%) | 83 (13.9%) | 115 (17.5%) |  | 47 (19.0%) | 67 (16.8%) |  |
|  | Not Completed high school or GED/Don't Know | 48 (3.8%) | 26 (4.4%) | 22 (3.3%) |  | 7 (2.8%) | 15 (3.8%) |  |
| **Smoking status** | Never smoked on permanent basis | 470 (37.5%) | 322 (53.9%) | 148 (22.5%) |  | 74 (30.0%) | 69 (17.3%) |  |
|  | Past smoker, quit more than a year ago | 144 (11.5%) | 79 (13.2%) | 65 (9.9%) | <0.001 | 32 (13.0%) | 30 (7.5%) | <0.001 |
|  | Past smoker, quit less than a year ago | 114 (9.1%) | 34 (5.7%) | 80 (12.2%) |  | 40 (16.2%) | 39 (9.8%) |  |
|  | Yes, some days | 328 (26.2%) | 78 (13.1%) | 250 (38.1%) |  | 81 (32.8%) | 167 (41.9%) |  |
|  | Yes, every day | 168 (13.4%) | 65 (10.9%) | 103 (15.7%) |  | 12 (4.9%) | 91 (22.8%) |  |
|  | Do not remember/Unsure | 30 (2.4%) | 19 (3.2%) | 11 (1.7%) |  | 8 (3.2%) | 3 (0.8%) |  |
| **Flu vaccine last year** | No | 580 (46.3%) | 361 (60.5%) | 219 (33.3%) |  | 131 (53.0%) | 85 (21.3%) |  |
|  | Yes | 587 (46.8%) | 186 (31.2%) | 401 (61.0%) | <0.001 | 103 (41.7%) | 296 (74.2%) | <0.001 |
|  | Do not remember/Unsure | 87 (6.9%) | 50 (8.4%) | 37 (5.6%) |  | 13 (5.3%) | 18 (4.5%) |  |

* Mechanical Ventilation from total hospitalized
