# Supplementary material for: Assessing Associations Between COVID-19 Symptomology and Adverse Outcomes After Piloting Crowdsourced Data Collection: Cross-sectional Survey Study
Source: JMIR Form Res. 2022 Dec 6;6(12):e37507. doi: 10.2196/37507 (PMC9746676; doi:10.2196/37507)
Supplement: Multimedia Appendix 6 [file formative_v6i12e37507_app6.docx]

**Multimedia Appendix 6.** Chronic condition characteristics of the study participants.
