# Supplementary material for: Assessing Associations Between COVID-19 Symptomology and Adverse Outcomes After Piloting Crowdsourced Data Collection: Cross-sectional Survey Study
Source: JMIR Form Res. 2022 Dec 6;6(12):e37507. doi: 10.2196/37507 (PMC9746676; doi:10.2196/37507)
Supplement: Multimedia Appendix 8 [file formative_v6i12e37507_app8.docx]

**Multimedia Appendix 8.** Associations between symptoms and adverse COVID-19 outcomes adjusted for sociodemographic factors and chronic conditions (multivariate logistic regression).

| **Characteristics** | **Hospitalization** | | | **Mechanical Ventilation** | | |
| --- | --- | --- | --- | --- | --- | --- |
|  |  | | |  | | |
|  | **OR** | **95% CI** | **P Value** | **OR** | **95% CI** | **P Value** |
|  |  |  |  |  |  |  |
| **Gender** (Female) | 1.00 |  |  |  |  |  |
| Male | 1.02 | (0.74-1.4) | 0.915 | 0.99 | (0.63-1.54) | 0.964 |
| **Age groups** (18 - 24) | 1.00 |  |  |  |  |  |
| 25 - 34 | 2.50 | (1.24-5.22) | 0.012 | 1.57 | (0.46-5.47) | 0.472 |
| 35 - 44 | 2.49 | (1.21-5.31) | 0.016 | 2.46 | (0.71-8.78) | 0.157 |
| 45 - 54 | 2.67 | (1.25-5.92) | 0.013 | 3.37 | (0.95-12.39) | 0.062 |
| 55 - 64 | 5.73 | (2.56-13.33) | <0.001 | 2.42 | (0.64-9.46) | 0.195 |
| 65 - 74 | 3.69 | (1.41-9.97) | 0.009 | 2.97 | (0.61-15.15) | 0.181 |
| 75 or older | 12.22 | (2.68-64.01) | 0.002 | 0.67 | (0.08-4.95) | 0.698 |
| less then 18 | 0.36 | (0.01-3.75) | 0.450 | NA |  |  |
| **Race** (White) | 1.00 |  |  |  |  |  |
| Black/African American | 0.80 | (0.47-1.33) | 0.383 | 1.42 | (0.63-3.22) | 0.396 |
| Asian American | 0.31 | (0.12-0.77) | 0.014 | 2.28 | (0.37-18.11) | 0.400 |
| Native American/American Indian/Alaska Native | 0.31 | (0.08-1.11) | 0.078 | 0.84 | (0.17-4.89) | 0.839 |
| Multiracial/Other | 0.44 | (0.15-1.18) | 0.114 | 2.98 | (0.4-27.09) | 0.304 |
| **Ethnicity** (Not Hispanic or Latino) | 1.00 |  |  |  |  |  |
| Hispanic or Latino | 1.19 | (0.77-1.86) | 0.436 | 1.68 | (0.93-3.06) | 0.088 |
| **Yearly income** ($75,000 or more) | 1.00 |  |  |  |  |  |
| $50,000 to $74,999 | 0.84 | (0.5-1.43) | 0.525 | 1.36 | (0.65-2.84) | 0.406 |
| $35,000 to $49,999 | 1.00 | (0.57-1.75) | 0.998 | 0.85 | (0.4-1.79) | 0.666 |
| $15,000 to $34,999 | 1.56 | (0.9-2.69) | 0.112 | 1.20 | (0.57-2.51) | 0.633 |
| $0 to $14,499 | 1.93 | (1-3.77) | 0.050 | 1.15 | (0.46-2.87) | 0.757 |
| **Education** (Grade 12/Completed high school or GED) | 1.00 |  |  |  |  |  |
| Some college/Associates Degree/Technical Degree | 0.71 | (0.37-1.36) | 0.295 | 1.48 | (0.49-4.48) | 0.484 |
| Bachelor's Degree | 2.57 | (1.43-4.66) | 0.002 | 1.68 | (0.65-4.46) | 0.290 |
| Any post graduate studies | 1.79 | (0.9-3.6) | 0.101 | 1.90 | (0.65-5.66) | 0.240 |
| Not Completed high school or GED/Don't Know | 1.55 | (0.52-4.92) | 0.442 | 2.13 | (0.47-10.71) | 0.340 |
| **Smoking status** (Never smoked on permanent basis) | 1.00 |  |  |  |  |  |
| Past smoker, quit more than a year ago | 1.19 | (0.7-2.02) | 0.523 | 1.14 | (0.53-2.49) | 0.734 |
| Past smoker, quit less than a year ago | 3.39 | (1.88-6.24) | <0.001 | 0.81 | (0.39-1.69) | 0.575 |
| Yes, some days | 3.41 | (2.2-5.33) | <0.001 | 1.60 | (0.9-2.89) | 0.113 |
| Yes, every day | 2.06 | (1.18-3.61) | 0.012 | 3.53 | (1.45-9.1) | 0.007 |
| **Flu vaccine last year** (No) | 1.00 |  |  |  |  |  |
| Yes | 3.09 | (2.18-4.41) | <0.001 | 3.65 | (2.29-5.89) | <0.001 |
| **Chronic Conditions** |  |  |  |  |  |  |
| Depression | 1.77 | (1.18-2.67) | 0.006 | 1.49 | (0.89-2.49) | 0.130 |
| Hypertension | 1.09 | (0.73-1.64) | 0.674 | 0.81 | (0.48-1.34) | 0.406 |
| Obesity | 1.59 | (0.96-2.67) | 0.074 | 1.18 | (0.65-2.16) | 0.589 |
| Asthma | 3.83 | (2.22-6.78) | <0.001 | 1.18 | (0.64-2.18) | 0.603 |
| Alcohol or substance use disorder | 0.75 | (0.42-1.37) | 0.346 | 0.67 | (0.33-1.34) | 0.254 |
| Diabetes, uncomplicated | 2.66 | (1.46-4.96) | 0.002 | 1.14 | (0.6-2.21) | 0.689 |
| Mental Illness | 0.64 | (0.33-1.23) | 0.178 | 1.32 | (0.55-3.23) | 0.541 |
| Anemia | 1.13 | (0.51-2.54) | 0.761 | 1.13 | (0.42-3.2) | 0.810 |
| Migraines | 0.76 | (0.39-1.47) | 0.412 | 0.86 | (0.3-2.47) | 0.772 |
| Weight loss | 2.05 | (0.98-4.48) | 0.064 | 1.70 | (0.76-3.95) | 0.206 |
| High Cholesterol | 0.31 | (0.13-0.7) | 0.006 | 0.47 | (0.12-1.74) | 0.267 |
| Ulcer | 0.68 | (0.34-1.35) | 0.267 | 0.76 | (0.32-1.81) | 0.538 |
| Thyroid Problems | 0.40 | (0.15-1.07) | 0.068 | 2.80 | (0.72-12.28) | 0.149 |
| Severe Allergy | 1.04 | (0.45-2.5) | 0.921 | 0.53 | (0.19-1.45) | 0.216 |
| Lung/Respiratory Disease | 1.65 | (0.59-5.16) | 0.364 | 2.99 | (0.85-13.03) | 0.110 |
| Cancer | 0.93 | (0.31-2.88) | 0.896 | 1.63 | (0.42-7.5) | 0.501 |
| Reflux/GERD | 0.24 | (0.07-0.73) | 0.017 | 0.25 | (0.02-3.05) | 0.284 |
| Diabetes, complicated | 1.45 | (0.52-4.33) | 0.489 | 2.71 | (0.77-10.25) | 0.127 |
| Bladder Problems | 5.51 | (1.28-27.13) | 0.028 | 0.53 | (0.11-2.64) | 0.426 |
| Drug abuse | 0.52 | (0.12-2.13) | 0.363 | 0.38 | (0.06-2.69) | 0.310 |
| Autoimmune problems | 0.63 | (0.14-2.67) | 0.529 | 3.14 | (0.25-138.83) | 0.454 |
| Bowel Disease | 6.24 | (0.99-42.44) | 0.055 | 0.41 | (0.05-3.3) | 0.401 |
| Heart Pain/Angina | 4.40 | (0.95-25.02) | 0.073 | 1.05 | (0.19-6.75) | 0.957 |
| Psychoses | 0.24 | (0.06-1) | 0.041 | 7.25 | (1.02-81.73) | 0.066 |
| Congestive heart failure | 2.68 | (0.54-15.65) | 0.245 | 2.17 | (0.31-19.49) | 0.455 |
| Seizures/Convulsions | 0.79 | (0.13-5.11) | 0.806 | 0.32 | (0.04-4.27) | 0.331 |
| Osteoporosis | 1.51 | (0.23-14.02) | 0.693 | 4.80 | (0.57-80.58) | 0.196 |
| Other mental health condition | 0.71 | (0.06-5.19) | 0.759 | 0.17 | (0-41.04) | 0.389 |
| Other chronic condition | 0.73 | (0.33-1.61) | 0.428 | 1.45 | (0.51-4.53) | 0.499 |
| **Covid-19 Symptoms** |  |  |  |  |  |  |
| Abdominal pain | 2.02 | (1.15-3.59) | 0.016 | 0.91 | (0.45-1.86) | 0.804 |
| Bladder pain | 3.20 | (1.25-9.3) | 0.022 | 2.59 | (0.95-7.7) | 0.073 |
| Chest discomfort, tightness or pressure | 0.89 | (0.55-1.42) | 0.614 | 0.83 | (0.43-1.62) | 0.582 |
| Chills | 0.72 | (0.46-1.13) | 0.149 | 1.20 | (0.63-2.32) | 0.576 |
| Confusion | 2.18 | (1.06-4.56) | 0.036 | 0.93 | (0.37-2.38) | 0.870 |
| Cough with sputum | 2.60 | (1.77-3.86) | <0.001 | 0.72 | (0.44-1.16) | 0.173 |
| Cramping legs | 1.00 | (0.27-3.88) | 1.000 | 3.19 | (0.42-30.14) | 0.288 |
| Diarrhea | 0.52 | (0.26-1.04) | 0.066 | 0.27 | (0.09-0.75) | 0.013 |
| Dizziness | 1.72 | (0.93-3.22) | 0.085 | 0.63 | (0.28-1.39) | 0.253 |
| Dry Cough | 1.32 | (0.93-1.86) | 0.121 | 1.10 | (0.68-1.8) | 0.700 |
| Dry skin | 2.39 | (0.86-7.06) | 0.102 | 1.21 | (0.45-3.34) | 0.709 |
| Dry eyes | 0.61 | (0.22-1.72) | 0.338 | 0.83 | (0.28-2.52) | 0.741 |
| Fever > 100.4 F or > 38 C | 1.50 | (1.04-2.17) | 0.030 | 0.95 | (0.58-1.58) | 0.849 |
| Fever but do not know exact temperature | 1.59 | (0.87-2.89) | 0.132 | 1.56 | (0.68-3.64) | 0.296 |
| General lack of energy or malaise | 0.18 | (0.09-0.35) | <0.001 | 2.27 | (0.65-8.11) | 0.199 |
| Headaches | 1.29 | (0.87-1.92) | 0.209 | 1.03 | (0.62-1.72) | 0.899 |
| Hair Loss | 0.53 | (0.09-3.12) | 0.481 | 0.25 | (0.03-2.58) | 0.198 |
| Hoarseness | 3.03 | (0.95-9.15) | 0.054 | 0.66 | (0.12-3.54) | 0.635 |
| Joint aches | 0.64 | (0.33-1.22) | 0.178 | 0.41 | (0.15-1.11) | 0.083 |
| Loss of ability to smell | 0.93 | (0.55-1.55) | 0.770 | 0.25 | (0.12-0.51) | <0.001 |
| Loss of ability to taste | 1.13 | (0.77-1.66) | 0.530 | 0.84 | (0.51-1.39) | 0.501 |
| Loss of appetite | 1.57 | (0.99-2.49) | 0.054 | 2.07 | (1.09-4.02) | 0.028 |
| Muscle aches | 0.71 | (0.47-1.07) | 0.102 | 0.76 | (0.43-1.33) | 0.336 |
| Nausea | 0.89 | (0.57-1.39) | 0.596 | 0.75 | (0.43-1.33) | 0.328 |
| Rhinorrhea | 0.37 | (0.09-1.47) | 0.158 | 1.78 | (0.24-21.15) | 0.601 |
| Runny or stuffy nose | 0.51 | (0.33-0.78) | 0.002 | 1.09 | (0.58-2.05) | 0.798 |
| Seizure | 3.98 | (0.82-24.7) | 0.105 | 0.61 | (0.11-3.68) | 0.569 |
| Shortness of breath | **2.75** | (1.8-4.23) | <0.001 | 1.50 | (0.86-2.64) | 0.158 |
| Skin rash | 3.70 | (0.96-16.67) | 0.070 | 0.49 | (0.14-1.73) | 0.265 |
| Sneezing | 1.48 | (0.99-2.21) | 0.054 | 0.41 | (0.24-0.69) | 0.001 |
| Sore throat | 0.94 | (0.64-1.37) | 0.739 | 0.79 | (0.48-1.33) | 0.377 |
| Sputum production | 1.26 | (0.35-4.38) | 0.724 | 0.70 | (0.06-6.31) | 0.764 |
| Stomach cramps | 1.16 | (0.39-3.37) | 0.791 | 1.23 | (0.32-4.96) | 0.764 |
| Tiredness or Fatigue | 0.41 | (0.24-0.69) | 0.001 | 2.36 | (1.04-5.44) | 0.042 |
| Vomiting | 1.85 | (0.97-3.6) | 0.065 | 2.68 | (1.3-5.71) | 0.009 |
| Weakness | 1.12 | (0.72-1.74) | 0.621 | 0.84 | (0.47-1.51) | 0.556 |
| Altered consciousness | 1.01 | (0.28-3.39) | 0.986 | 1.55 | (0.15-19.22) | 0.724 |
| Other symptoms | 0.92 | (0.05-6.76) | 0.944 | NA |  |  |
