# Supplementary material for: Assessing Associations Between COVID-19 Symptomology and Adverse Outcomes After Piloting Crowdsourced Data Collection: Cross-sectional Survey Study
Source: JMIR Form Res. 2022 Dec 6;6(12):e37507. doi: 10.2196/37507 (PMC9746676; doi:10.2196/37507)
Supplement: Multimedia Appendix 9 [file formative_v6i12e37507_app9.docx]

**Multimedia Appendix 9.** Comparison of our findings with those of systematic review and meta-analysis studies regarding the association between COVID-19 symptoms and adverse outcomes.

| Study author | Barek 2020 | Li 2021 | Booth 2021 | Current study | Agreement between current study with at least one previous study |
| --- | --- | --- | --- | --- | --- |
| Study design | Meta-analysis | Systematic review and meta‐analysis | Systematic review | Cross-sectional survey |  |
| Sample size | N=10,014 | N=281,461 | N=17,860,001 | N=1,254 |  |
| **Covid-19 symptoms** | ***Significantly associated with the Covid -19 adverse outcomes*** | | | |  |
| Abdominal pain | Yes | Yes | No | Yes | 1 |
| Altered consciousness | NA | NA | NA | No | New |
| Bladder pain | NA | NA | NA | Yes | New |
| Anorexia | Yes | No | No | NA | NA |
| Chest tightness | Yes | Yes | No | No | 1 |
| Chills | NA | No | Yes | No | 1 |
| Confusion | NA | NA | NA | No | New |
| Cough | Yes | No | No | Yes | 1 |
| Cramping legs | NA | NA | NA | No | New |
| Diarrhea | Yes | No | No | No | 1 |
| Dizziness | No | No | No | No | 1 |
| Dyspnea | Yes | NA | Yes | NA | NA |
| Dry eyes | NA | NA | NA | Yes | New |
| Dry skin | NA | NA | NA | Yes | New |
| Fatigue | Yes | Yes | No | Yes | 1 |
| Fever | Yes | No | No | Yes | 1 |
| Hair Loss | NA | NA | NA | Yes | New |
| Headache | No | No | No | No | 1 |
| Hemoptysis | Yes | NA | NA | NA | NA |
| Hoarseness | NA | NA | NA | No | New |
| Joint aches | NA | NA | NA | No | New |
| Loss of smell/taste | NA | NA | No | No | 1 |
| Loss of appetite | NA | NA | NA | Yes | New |
| Malaise | NA | No | NA | No | 1 |
| Myalgia | No | No | Yes | No | 1 |
| Nausea | No | No | Yes | No | 1 |
| Pharyngalgia | NA | NA | No | NA | NA |
| Rhinorrhea | NA | No | No | No | 1 |
| Seizure | NA | NA | NA | No | New |
| Shortness of breath | NA | Yes | NA | Yes | 1 |
| Skin rash | NA | NA | NA | Yes | New |
| Sneezing | NA | NA | NA | No | New |
| Sore throat | No | NA | NA | No | 1 |
| Sputum production | No | NA | Yes | No | 1 |
| Stomach cramps | NA | NA | NA | No | New |
| Vomiting | No | Yes | No | Yes | 1 |
| **Summary of symptoms** |  |  |  |  |  |
| Total measured symptoms | 16 | 16 | 18 | 32 |  |
| Symptoms associated with adverse outcomes | 9 | 5 | 5 | 11 |  |
| Agreement between current study and previous studies |  |  |  |  | 18 (100%) |
| New measured symptoms |  |  |  |  | 14 |
| Overlapping symptoms associated with adverse outcomes |  |  |  |  | 5 |
| New symptoms associated with adverse outcomes |  |  |  |  | 6 |
